# Job motivation and associated factors among healthcare professionals serving in primary hospitals in the Central Gondar Zone, Northwest Ethiopia: a mixed-methods approach

**DOI:** 10.3389/fpubh.2025.1494447

**Published:** 2025-02-19

**Authors:** Mandefro Tadesse Legesse, Habtamu Wagnew Abuhay, Nigusu Worku, Endalkachew Dellie

**Affiliations:** ^1^Health Systems and Policy Program, Institute of Public Health, College of Medicine and Health Sciences, University of Gondar, Gondar, Ethiopia; ^2^Department of Epidemiology and Biostatistics, Institute of Public Health, College of Medicine and Health Sciences, University of Gondar, Gondar, Ethiopia; ^3^Department of Health Systems and Policy, Institute of Public Health, College of Medicine and Health Sciences, University of Gondar, Gondar, Ethiopia

**Keywords:** job motivation, healthcare professional, primary hospital, mixed method approach, Ethiopia

## Abstract

**Background:**

Low performance in health facilities is associated mostly with poor healthcare worker motivation in the workplace, and this burden is dominant in developing countries such as Ethiopia. Therefore, this study aimed to assess job motivation and associated factors among healthcare professionals in Central Gondar Zone primary hospitals in Northwest Ethiopia.

**Methods:**

An institutionally based cross-sectional study with a qualitative component was conducted at Central Gondar Administrative Zone primary hospitals in the Amhara National Regional State, Ethiopia. A total of 396 healthcare professionals with more than six months of experience at primary hospitals in the Central Gondar Zone participated. Multiple logistic regression was fitted, and Odds ratio (OR) with 95% CIs was used to measure the strength of associations and variables with a *p*-value of <0.05 were considered significantly associated with the outcome. Thematic analysis was performed for the qualitative part.

**Results:**

A total of 41.81% of healthcare professionals with a 95% Cl [36.9, 46.6] had good job motivation. A degree of educational level (AOR = 1.84, 95% CI [1.178, 2.874]), being satisfied with training opportunities (AOR = 1.92, 95% CI [1.201, 3.083]), and having a written job description (AOR = 2.58, 95% CI [1.497, 4.460]) were significantly associated with job motivations.

**Conclusion:**

Healthcare professionals’ job motivation status was relatively low. In addition, having a degree in educational level, being satisfied with training opportunities, and having a written job description were significant positive predictors of good job motivation. Additionally, the thematic analysis of qualitative data identified both short- and long-term training opportunities and the lack of clear, updated job descriptions, both of which significantly impacted job motivation.

## Introduction

Motivation can be defined as an “individual’s degree of willingness to exert and maintain an effort toward organizational goals” (1). Health is maintained through the efforts of healthcare professionals who adhere to established principles and practices. In the workplace, human resources play a pivotal role in ensuring the functionality of the healthcare system (2). The motivation of healthcare professionals is strongly influenced by their connection to and interaction with their work environment, which directly impacts their performance and the quality of care provided (3).

Healthcare professionals who are not motivated well might have a detrimental impact on individuals, institutions, and the broader healthcare system. Many countries are currently experiencing a scarcity of trained healthcare professionals; the loss of healthcare professionals has major consequences for community and country health. Maintaining the motivation of healthcare professionals aids the seamless operation of the complete healthcare system (4, 5).

Globally, low job motivation was recorded as the second most important health expert problem in the world (6). The African region is experiencing both a high burden of disease and the lowest density of healthcare workers (5). According to surveys on job motivation at Gaza Hospital, approximately 66% of healthcare workers were motivated at their jobs (7). The magnitude of healthcare professionals’ job motivation in Kenya, and Ethiopia were 35.4, and 41.7%, respectively, who were demotivated to provide routine healthcare to clients (8, 9).

Poor workplace motivation leads to increased staff turnover, reduced levels of engagement, and lower productivity. Job motivation is affected by various factors, such as sociodemographic factors such as age and sex, as well as factors related to the work environment, professional advancement, training, supervision, and rewards, which are the most important factors associated with job motivation (10–12).

Although few studies have been conducted to ascertain job motivation and associated factors among healthcare professionals, most of them have focused only on quantitatively (9, 11). Some of these studies were conducted five years before, which is not representative of the current situation of job motivation among healthcare professionals, where many human resource guidelines regarding salary and incentive packages were changed. Therefore, the purpose of this study was to assess job motivation and associated factors among healthcare professionals in the Central Gondar Zone primary hospital, Amhara National Regional States, Northwest Ethiopia.

## Methods

### Study design and settings

An institution-based cross-sectional quantitative study, supplemented by a qualitative component, was conducted from May 24 to June 24, 2022. The qualitative data were collected concurrently using a phenomenological approach and were analyzed and integrated during the interpretation phase to provide a comprehensive understanding of the findings. The study was conducted at a primary hospital in the Central Gondar Administrative Zone of the Amhara National Regional State, one of the 12 zones in the region. The zone comprises 15 districts and four city administrations, totaling 19 districts with a population of 2,307,773. Among these districts, 843 healthcare workers are employed at primary hospitals serving the catchment area (13). Gondar City, the seat of the Central Gondar Zone, is located approximately 750 kilometers north of Addis Ababa, the capital of Ethiopia. Nine primary hospitals were included in this study: Delgi Hospital, Wogera Hospital, Arbaya Hospital, Tegede Hospital, Dembia Hospital, Sanja Hospital, Shawra Hospital, Aykel Hospital, and Guhala Hospital.

### Population

All healthcare professionals working in primary hospitals within the Central Gondar Zone, Northwest Ethiopia were the source and study population. Healthcare professionals with more than six months of work experience were included in the study. However, healthcare professionals employed on a contract basis or working as volunteer staff were excluded.

### Sample size and sampling techniques

The required sample size was calculated using both single and double population proportion formulas, and the largest sample size among them was selected. The overall proportion of job motivation among healthcare professionals was 0.586% (14), with a 95% confidence level (Z_α/2_ = 1.96) and a margin of error = 5%. The calculation indicated a sample size of 373. After adding a 10% nonresponse rate, the total sample size was estimated to be 411 healthcare professionals.

For the qualitative study, the purposive sampling method was applied to select six study participants up to the level of saturation reached.

The Central Gondar Zone comprises nine primary hospitals, which collectively employ a total of 843 healthcare professionals. For this study, the sample size was distributed proportionally based on the number of healthcare professionals in each hospital. From this total, 411 healthcare professionals who met the inclusion criteria were selected. A computer-generated simple random sampling method was used to identify the final study participants, utilizing a sampling frame obtained from the human resource departments of all nine primary hospitals.

For the qualitative study, the purposive sampling method was applied to select six key informants up to the level of saturation reached, and six key informants (one hospital manager, two medical directors, one hospital matron, one case manager, and one case team coordinator) were selected.

### Variables and measurements

The dependent variable of the study was job motivation. The independent variables were sociodemographic variables (age, sex, marital status, religion, educational status, profession, work experience, salary), institutional factors (supervision, relationship with a coworker, fair treatment, reward and recognition, availability of equipment, working conditions, training opportunities, performance evaluation, job description, and career and professional development), and external factors (hospital location, transportation).

#### Job motivation

The willingness to exert different levels of effort toward achieving organizational goals (1). It is a dependent variable that was measured via 23 items in 7 motivational measuring dimensions (10). These are the general motivational domain (3 items), burnout domain (2 items), job satisfaction domain (3 items), intrinsic satisfaction domain (3 items), organizational commitment domain (5 items), conscientiousness domain (4 items), and timelines dimension (3 items) of a five-point Likert rating scale, with endpoints ranging from 1 = strongly disagree to 5 = strongly agree.

#### Good motivation

Score above or equal to the mean from a cumulative of 23 motivation measuring questions of the study populations (15, 16).

Poor motivation scores below the mean from a cumulative of 23 motivation measuring questions of the study populations (15, 16).

#### Availability of equipment

Availability of equipment refers to the presence of medical supplies and medical equipment in the hospital for patient care. It is measured when an individual responds greater than three on a Likert scale question (17).

#### Working condition

Working conditions refer to the working environment, including infrastructure such as the availability of electricity and access to clean water. A satisfied working condition is defined and measured as when an individual responds greater than three on a Likert scale (17).

### Data collection tools and procedures

The structured English version of the data collection questionnaires was adapted and modified based on other previous similar studies (10, 17). The questionnaire consists of four parts: the first part covers sociodemographic characteristics, the second part addresses institutional factors, the third part focuses on external factors, and the fourth part includes questions designed to measure job motivations. The data collection tool for the qualitative parts of the tape recorder and interview guide was used for key informant interviews (KIIs), such as those with a hospital manager, medical director, case manager, matron, and case team coordinator, which were conducted by using an interview guide, and the audio was recorded. Then, the data were transcribed in the Amharic language, translated into English, and entered into open code version 4.03 software for thematic analysis of the qualitative data.

Eight BSc nurses were recruited as data collectors to complete a self-administered questionnaire under the supervision of four supervisors with qualifications of Master of Public Health (MPH). Besides, the qualitative interviews were conducted by one trained data collector in a private and quiet setting to ensure confidentiality. Each interview lasted approximately one to two hours, depending on the depth of responses.

### Data processing and analysis

The data were first checked for completeness and consistency. The data were subsequently entered into EpiData version 4.6 and exported to STATA version 14.0 for quantitative analysis. For qualitative analysis, open code version 4.03 software was used. Before analysis, the data were edited, verified, cleaned, coded, and merged as necessary to make them suitable for quantitative analysis, and the data were transcribed word by word (verbatim) and translated into English for qualitative analysis. Descriptive statistics for categorical variables were presented as percentages and frequencies. A continuous variable was also described using the mean, and the standard deviation was calculated and additionally explored through graphical techniques. Binary logistic regression was performed, and assumptions for model fitness were checked. We conducted bivariable analyses to examine the relationship between each independent variable and the dependent variable (job motivation). Variables with *p*-values ≤0.2 in the bi-variable analyses were included in the multi-variable logistic regression. The degree of association between independent and dependent variables was assessed by using an adjusted odds ratio (AOR) with a 95% confidence interval (Cl) as a measure of association. Variables with a *p*-value of less than 0.05 in the multivariable model were considered statistically significantly associated with the outcome. The goodness of fit test of the Hosmer–Lemeshow test of the *p*-value was 0.80, where the data was adequately fitted with the models.

## Results

### Sociodemographic characteristics of the participants

A total of 397 participants responded to the interviewer-administered questionnaire with a response rate of 96.6%. The mean and SD ages of the study participants were 29.43 and 4.61 years, respectively. The majority of the study participants (72.29%) were males. The majority (57.93%) of the respondents had a first degree, and one hundred forty (35.26%) of the study participants were nurses. Three hundred eighty-one (95.97%) of the study participants were orthodox Christians, and 177 (44.58%) of the respondents had 6 years or more of work experience. With respect to marital status, two hundred six (51.89%) of the participants were married (Table 1).

**Table 1:** Sociodemographic characteristics of healthcare professionals in Central Gondar Zone primary hospitals.

Characteristics	Categories	Frequency	Percent (%)
Age	< =25 years	56	14.11
	26–30 years	237	59.70
	>30 years	104	26.20
Sex	Male	287	72.29
	Female	110	27.71
Educational status	Diploma	150	37.78
	Degree	230	57.93
	Second degree	17	4.28
Profession	Medical doctor	46	11.59
	Diploma/BSc Nurse	140	35.26
	Laboratory technician/technologist	46	11.59
	Pharmacy technician/technologist	49	12.34
	Diploma/BSc Midwifery	54	13.60
	Other*	62	15.62
Marital status	Single	191	48.11
	Married	206	51.89
Religion	Orthodox	381	95.97
	Muslim	13	3.27
	Protestant	3	0.76
Work experience	< 2 Years	91	22.92
	2–5 year	129	32.49
	6 years and above	177	44.58
Salary	<4,609	125	31.49
	4,610–7,071	213	53.65
	> = 7,072	59	14.86

*radiographer, health officer, health informatics, environmental health, emergency obstetric surgeon, BSc anesthetics.

### Institutional and external factors

Two hundred thirty-one (58.19%) of the study participants were not satisfied with the training opportunities. Two hundred two (50.88%) of the study participants reported that there was not sufficient equipment, 348 (87.66%) of the respondents were satisfied with coworker relationships, and 221 (55.67%) of the respondents were satisfied with working conditions. Regarding job descriptions, 283 (71.28%) of the respondents reported that they did not have a written job description.

Two hundred forty-two (60.96%) of the participants were satisfied with the performance evaluation, whereas 253 (63.73%) of the respondents were dissatisfied with unfair treatment. The majority (56.68%) of the respondents were dissatisfied with supervision, and 207 (52.14%) of the respondents were dissatisfied with their careers and professional development. The majority (69.77%) of the respondents were not satisfied with the rewards and recognition. Transportation services were inconvenient for most participants (83.12%), and facility locations were inconvenient for nearly three-fourths (73.55%) of the respondents (Table 2).

**Table 2:** Institutional and external factors related to healthcare professionals working in Central Gondar Zone primary hospitals.

Characteristics	Categories	Frequency	Percent (%)
Training opportunity	Not-satisfied	231	58.19
	Satisfied	166	41.81
Availability of equipment	Yes	195	49.12
	No	202	50.88
Relationship with coworker	Not-satisfied	49	12.34
	Satisfied	348	87.66
Fair treatment	Yes	144	36.27
	No	253	63.73
Working condition	Satisfied	221	55.67
	Not-satisfied	176	44.33
Supervision	Not-satisfied	225	56.68
	Satisfied	172	43.32
Job description	Having	114	28.72
	Not having	283	71.28
Performance evaluation	Not-satisfied	155	39.04
	Satisfied	242	60.96
Career and professional development	Not-satisfied	207	52.14
	Satisfied	190	47.86
Recognition and reward	Not-satisfied	277	69.77
	Satisfied	120	30.23
Transportation	Convenient	67	16.88
	Inconvenient	330	83.12
Hospital location	Convenient	105	26.45
	Inconvenient	292	73.55

### Healthcare professionals’ job motivation

**Figure 1:**
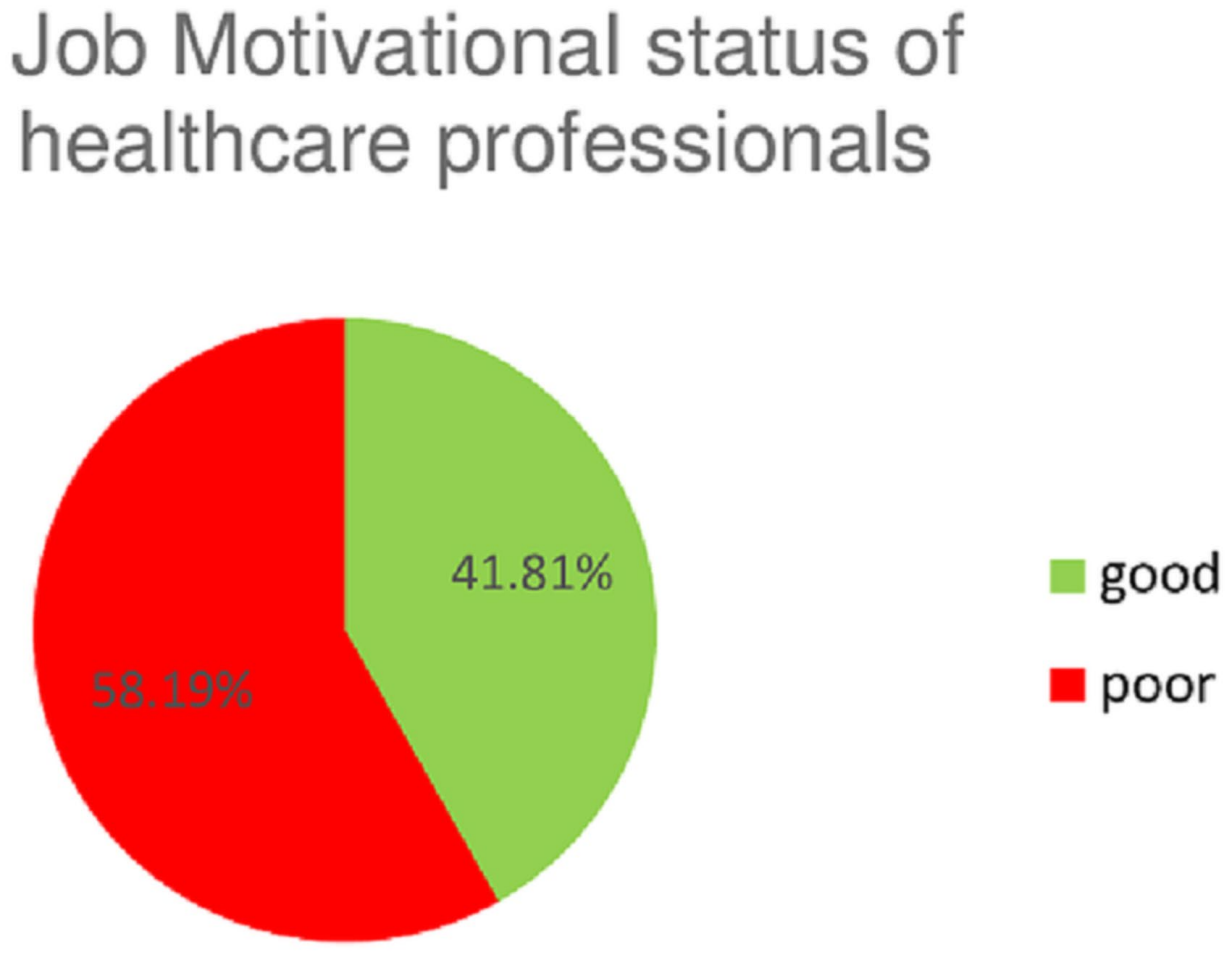
Healthcare professional job motivational status in Central Gondar Zone primary hospitals, 2022.

Among the total study participants, a healthcare professional’s 23 items of questionnaire within seven dimensions were used to assess job motivation status. The percentage of healthcare professionals’ good motivation status was 41.81%, with a 95% CI [36.9, 46.6%] (Figure 1).

### Factors associated with job motivation

In a binary logistic regression analysis, the majority of sociodemographic and institutional factors were associated with job motivation. Variables with a *p*-value <=0.2 in the bi-variable analysis were considered for the multivariable analysis. Accordingly, educational status, training opportunities, availability of equipment, coworker relationships, fair treatment, working conditions, job description, transportation, hospital location, performance evaluation, reward, and recognition by managers were significantly associated with job motivation and candidates for multivariable logistic regression analysis. Correspondingly, in the multivariable analysis, only educational status, training opportunities, and job description were significantly associated with job motivation.

Accordingly, respondents who had a degree educational status were 1.84 times more likely to be motivated with their job than diploma holders were (AOR = 1.84, 95% CI = [1.178, 2.874]). Similarly, participants who were satisfied with the training opportunity were 1.92 times more likely to be motivated than their counterparts (AOR 1.92, 95% CI [1.201, 3.083]).

In addition, those having a written job description were 2.5 times more likely to be motivated by their job than those who did not have a written job description (AOR = 2.58, 95% CI = [1.497, 4.460]) (Table 3).

**Table 3:** Bivariate and multivariate analyses of factors associated with job motivation in Central Gondar Zone primary hospitals.

Variables		Job motivation *n* (397)		COR (95%CI)	AOR (95% CI)	*P*-value
		Good motivation	Poor motivation			
Educational status	Diploma	74 (18.64)	76 (19.14)	1	1	
	Degree	86 (21.66)	144 (36.27)	1.63 [1.074, 2.473]	**1.84 [1.178, 2.874]**	**0.007**
	Second degree	6 (1.51)	11 (2.77)	1.78 [0.627, 5.075]	1.94 [0.646, 5.877]	0.236
Training Opportunity	Not-satisfied	115 (28.97)	116 (29.22)	1	1	
	Satisfied	51(12.85)	115(28.97)	2.23 [1.470, 3.397]	**1.92 [1.201, 3.083]**	**0.006**
Availability of equipment	Not available	75 (18.89)	127 (31.99)	1	1	
	Available	91 (22.92)	104 (26.20)	0.67 [0.452, 1.007]	1.09 [0.678, 1.762]	0.114
Relationship with coworker	Not satisfied	15 (3.78)	34 (8.56)	1	1	
	Satisfied	151 (38.04)	197 (49.62)	0.57 [0.302, 1.095]	0.77 [0.381, 1.562]	0.271
Fair treatment	No	94 (23.68)	159 (40.05)	1	1	
	Yes	72 (18.14)	72 (18.14)	0.59[0.390, 0.894]	0 0.84 [0.521, 1.369]	0.494
Working condition	Not satisfied	56(14.11)	120(30.23)	1	1	
	Satisfied	110 (27.71)	111 (27.96)	0.47 [0.311, 0.711]	0.74 [0.456, 1.228]	0.252
Performance evaluation	Not satisfied	54 (13.60)	101 (25.44)	1	1	
	Satisfied	112(28.21)	130(32.75)	0.62 [0.409, 0.940]	1.15 [0.694, 1.918]	0.180
Job description	Not having	139 (35.01)	144 (36.27)	1	1	
	Having	27 (6.80)	87 (21.91)	3.11 [1.904, 5.080]	**2.58 [1.497, 4.460]**	**0.001**
Transportation	Inconvenient	131 (33.00)	199 (50.13)	1	1	
	Convenient	35(8.82)	32(8.06)	0.60 [0.355, 1.020]	0.95 [0.522, 1.751]	0.086
Hospital location	Inconvenient	112 (28.21)	180 (45.34)	1	1	
	Convenient	54 (13.60)	51 (12.85)	0.58 [0.374, 0.921]	0.71 [0.430, 1.200]	0.207
Reward and recognition	Not satisfied	104 (26.20)	173(43.58)	1	1	
	Satisfied	62 (15.62)	58 (14.61)	0.56 [0.364, 0.866]	0.82 [0.495, 1.359]	0.143

COR: Crude odds ratio, CI: confidence interval, AOR: adjusted odds ratio, 1: reference category. Bold values indicate statistically significant results at *p* < 0.05 with a 95% CI.

### Qualitative findings

Two themes and four sub-themes were identified by the qualitative study findings. These themes are limited availability of training opportunities and poor job descriptions with regard to healthcare professional job motivations.

#### Theme 1: limited availability of training opportunities

Key informants highlighted that limited training opportunities negatively impact healthcare professionals’ motivation and performance.

##### Short-term training opportunities

Short-term training programs, essential for refreshing knowledge and improving performance, are scarce.

*“Short-term training opportunities are limited or not available.” (A 32-year-old medical director, KII 1)*

##### Long-term educational opportunities

Long-term programs like diplomas and master’s degrees are nearly non-existent, creating barriers to growth and reducing motivation.

*“The educational opportunities are too small and not proportional to the number of health professionals, which reduces motivation.” (A 32-year-old hospital matron, KII 4)*

#### Theme 2: poor job descriptions

Most informants noted that unclear and outdated job descriptions contribute to confusion and demotivation.

##### Lack of clarity

Job descriptions are often unclear, making it hard for professionals to understand their roles.

*“The job description lacks clarity; therefore, healthcare professionals cannot understand it completely.” (A 32-year-old hospital manager, KII 5)*

##### Outdated and underutilized

Job descriptions are not regularly updated or widely trusted, leading to low utilization and motivation.

*“Job descriptions are not regularly updated and are not used by professionals.” (A 33-year-old Case Team Coordinator, KII 3)*

## Discussion

Healthcare professionals are among the most vital resources in the healthcare delivery system. A motivated healthcare professional is highly important for an organization to achieve its objectives by increasing both service delivery and quality of care. Therefore, this study assessed job motivation and associated factors among healthcare professionals in Central Gondar Zone primary hospitals, Amhara National Regional States, Northwest Ethiopia.

In the present study, approximately 41.81% of healthcare professionals were motivated, with a 95% Cl [36.9, 46.6]. This finding is high compared with that of a study conducted in southwest Ethiopia, where 25.1% of healthcare professionals were motivated (17). However, this percentage is lower than that reported in a study conducted among healthcare professionals in the West Shoa Zone and West Amhara public hospitals, which reported that 63.6 and 58.6% of health professionals, respectively, were motivated in their jobs (11, 18). In addition, other studies in Zambia and Iran reported that 60–74 and 66% of healthcare professionals, respectively, were motivated (7, 19). The observed differences could be attributed to variations in the study populations. For instance, some of the referenced studies exclusively focused on nurses, whereas the current study included all categories of healthcare professionals. Additionally, contextual differences, such as variations across countries, regions, and types of healthcare facilities, may explain these discrepancies. Notably, most prior studies were conducted in specialized hospitals, while the present study was conducted in primary hospitals, which may influence healthcare professionals’ motivation levels.

According to our findings, healthcare professionals who have a degree education level had greater job motivation than diploma holders. This result is in line with studies performed in central Ethiopia and Nepal that showed that degree and above respondents had a higher motivation level than diploma holders (18, 20). However, a study performed in Jimma town, Southwest Ethiopia, revealed that nurses who were BSC degree holders and above were less likely to be motivated than those who were diploma holders (16). The similarity may be due to comparable educational systems and career structures, where degree-level education enhances knowledge, skills, and motivation. Higher educational attainment often equips individuals with advanced critical thinking, problem-solving abilities, and opportunities for career advancement, which can foster job satisfaction and motivation. The difference with the Jimma study could stem from population differences, as it focused solely on nurses, who may face unique job challenges, unlike the broader group of healthcare professionals in our study. Additionally, setting differences, with our study conducted at primary hospitals and theirs at public health centers, may explain the variation in resources, and management.

In this study, participants who were satisfied with training opportunities were 1.92 times more likely to be motivated than their counterparts. This result was similar to those of other studies performed at Jimma University, which reported that the presence of regular training opportunities increases the job motivation of healthcare professionals (17). In addition, studies performed in Kenya and Uganda have shown that training opportunities increased job motivation (8, 21). Training improves knowledge, skills, and self-confidence; helps employees understand how their work fits into their organization’s structure, mission, and goals; and can improve work quality and outcomes (22).

This study is supported by Herzberg’s intrinsic theory of motivation and Abraham Maslow’s self-esteem theory of motivation (23). This finding is also supported by Michael Armstrong’s learning and development theories: “Training is the systematic development of the knowledge, skills, and attitudes required by an individual to perform adequately a given task or job” (24). The possible similarity might be due to the use of a similar study population because the study was performed with all healthcare professionals, the study design, and data collection tools.

The qualitative findings from this study further highlight the critical issue of inadequate educational opportunities as a demotivating factor for healthcare professionals. *“The educational opportunities are too small and not proportional to the number of health professionals, which reduces motivation.” (A 32-year-old hospital matron, KII 4).*

This finding underscores the importance of continuous professional development as a key driver of motivation and job satisfaction in the healthcare sector. Access to adequate educational and training opportunities is essential for healthcare professionals to remain competent in their roles and to advance in their careers. Limited opportunities for training not only hinder skill development but also create a sense of stagnation among employees, leading to diminished morale, and job motivation.

In this study, healthcare professionals with a written job description were more likely to be motivated with their jobs than those who did not have a formal written job description. This result is also supported by another study performed among health workers at primary-level health facilities in rural Tanzania, which revealed that healthcare workers who had a job description had significantly higher motivation scores than those who had no job description (25). This could be due to the lack of a formal written job description leading to poor evaluations of the job, unclear progress of the work done, and decreased interest in work for healthcare professionals. It is difficult for an employee to have an accurate and complete understanding of his role (26).

Furthermore, the qualitative findings of this study highlight the significant role of clear job descriptions in supporting the motivation of healthcare professionals. A thirty-two years’ hospital manager reported that;

*“The job description lacks clarity; therefore, healthcare professionals cannot understand it completely.” (A 32-year-old hospital manager, KII 5)*. This observation underscores the critical challenge of role ambiguity in healthcare settings, which can negatively impact motivation and overall performance. Role clarity is an essential component of effective human resource management, particularly in healthcare, where the demands of the profession are inherently high-stakes.

The findings of this study are important for providing information for healthcare professionals, healthcare managers, policymakers, and human resource managers in hospitals, as low job motivation can be used as a signal of the quality of health services. In addition, it provides information about factors that are associated with job motivation, so it helps to minimize low motivation and maximize the effort of job motivation. The public health importance of this study is to increase the quality of service delivery in disease prevention and disease management by motivating healthcare professionals and identifying the factors most significantly associated with job motivation.

## Conclusion

In the current study, job motivation was relatively low compared with that reported in a previous similar study in Ethiopia. In addition, having a degree in education status, being satisfied with training opportunities, and having written job descriptions were positive significant predictors of good job motivation. In addition, qualitatively, most of the key informants agreed that healthcare professionals have low job motivation.

## Data Availability

The original contributions presented in the study are included in the article/supplementary material, further inquiries can be directed to the corresponding author.
